# ABCB4 missense mutations D243A, K435T, G535D, I490T, R545C, and S978P significantly impair the lipid floppase and likely predispose to secondary pathologies in the human population

**DOI:** 10.1007/s00018-017-2472-6

**Published:** 2017-02-20

**Authors:** Edward J. Andress, Michael Nicolaou, Farrell McGeoghan, Kenneth J. Linton

**Affiliations:** Centre for Cell Biology and Cutaneous Research, Blizard Institute, Barts and The London School of Medicine and Dentistry, Queen Mary University of London, 4 Newark Street, E1 2AT London, UK

**Keywords:** MDR3, Inflammatory liver disease, Sclerosing cholangitis, Biliary cirrhosis, Bile flow, Liver cancer, Cholestasis

## Abstract

Bile salts are natural detergents required to solubilise dietary fat and lipid soluble vitamins. They are synthesised in hepatocytes and secreted into the luminal space of the biliary tree by the bile salt export pump (BSEP), an ATP-binding cassette (ABC) transporter in the canalicular membrane. BSEP deficiency causes cytotoxic accumulation of bile salts in the hepatocyte that results in mild-to-severe forms of cholestasis. The resulting inflammation can also progress to hepatocellular cancer via a novel mechanism involving upregulation of proliferative signalling pathways. A second ABC transporter of the canalicular membrane is also critical for bile formation. ABCB4 flops phosphatidylcholine into the outer leaflet of the membrane to be extracted by bile salts in the canalicular space. These mixed micelles reduce the detergent action of the bile salts and protect the biliary tree from their cytotoxic activity. ABCB4 deficiency also causes cholestasis, and might be expected to cause cholangitis and predispose to liver cancer. Non-synonymous SNPs in *ABCB4* have now been described in patients with liver cancer or with inflammatory liver diseases that are known to predispose to cancer, but data showing that the SNPs are sufficiently deleterious to be an etiological factor are lacking. Here, we report the first characterisation at the protein level of six ABCB4 variants (D243A, K435T, G535D, I490T, R545C, and S978P) previously found in patients with inflammatory liver diseases or liver cancer. All significantly impair the transporter with a range of phenotypes exhibited, including low abundance, intracellular retention, and reduced floppase activity, suggesting that ABCB4 deficiency is the root cause of the pathology in these cases.

## Introduction

Cholesterol is a planar amphiphile comprising three cyclohexane rings (rings A to C) and one cyclopentane ring (ring D), with a hydroxyl group on the A ring and a hydrocarbon carbon tail on the D ring. It readily intercalates into the plasma membrane where the hydrophobic steroid rings and hydrocarbon tail intercalate between the acyl chains of the membrane lipids, and the hydroxyl interacts with their polar head groups. In the liver, cholesterol is catabolised primarily into bile acids. The simplest primary bile acid (chenodeoxycholic acid) has a second hydroxyl on the B ring and a carboxyl end to the hydrocarbon tail [1]. It is no longer planar and the axis of amphiphilicity extends the length of the molecule giving it a hydrophobic and a hydrophilic face. These features make bile acids extremely effective at solubilising dietary fat. The same features also cause bile acids to solubilise lipids from cell membranes and they are, therefore, inherently cytotoxic. On secretion from the hepatocyte across the canalicular membrane by the bile salt export pump (BSEP, ABCB11), monomeric bile acids pose a cytotoxic threat to the hepatocytes and cholangiocytes that line the biliary tree. Higher eukaryotes have therefore evolved mechanisms to protect the biliary tree from these hepatic metabolites. Excess phosphatidylcholine (PC) is flopped into the outer leaflet of the canalicular membrane of hepatocytes by a second ABC transporter (ABCB4). The PC is solubilised from the outer leaflet to form a mixed micelle that reduces the detergent activity (and, therefore, the cytotoxicity) of the bile acids in the canalicular space of the biliary tree. Cells that line the biliary tree also express the P-type ATPase ATP8B1 that functions in complex with CDC50. ATP8B1/CDC50 flips a different phospholipid (phosphatidylserine) in the opposite direction to the PC flopped by ABCB4. This is thought to preserve the lipid asymmetry of the hepatocyte and cholangiocyte membranes that line the biliary tree to resist the detergent activity of the bile acids. These protective mechanisms are known to be critical for the health of the biliary tree, because pedigree analysis shows definitively that mutations in *ATP8B1* and *ABCB4* cause two forms of fatal liver disease: Progressive Familial Intrahepatic Cholestasis (PFIC) types 1 and 3, respectively [2, 3]. These, and PFIC type 2 which is caused by *ABCB11*-deficiency [4], all result in end-stage liver disease in childhood, but the underlying mechanisms are different: in type 1, the membranes of the biliary tree are more susceptible to detergent damage; in type 2, damaging levels of bile acids accumulate in the hepatocyte; and in type 3, unquenched bile acids accumulate in the canalicular space.

Heterozygous and non-synonymous variants in these genes have been associated with a spectrum of cholestatic conditions, including intrahepatic cholestasis of pregnancy (ICP), benign recurrent intrahepatic cholestasis, susceptibility to drug-induced liver injury, and cholelithiasis (cholesterol gallstones (CG) which, specifically for ABCB4 SNPs, can develop into a syndromic condition termed low-phospholipid associated cholelithiasis (LPAC) in Europe [5] and Oriental Cholangiohepatitis in Japan [6]). Recently, *ABCB11* deficiency was also shown to be a driver of hepatocellular cancer in children and mice via a mechanism that lacks a mutational signature of typical cancer genes, but which has an inflammatory basis leading to genome instability and amplification of the MAPK signalling pathway [7]. Mice deficient in the PC-floppase ABCB4 (*mdr2* knock-out mice) do not fully develop PFIC type 3 because of the reduced toxicity of the rodent bile acid pool, but they do develop cholelithiasis [8] and inflammatory liver disease most similar to Sclerosing Cholangitis (SC) [9]. They also develop liver cancer [10, 11] via the same pathway of chronic inflammation that leads to genome instability and copy number gains in the MAPK signalling pathway [7]. *ABCB4* non-synonymous SNPs have been reported in human patients with liver cancer (encoding variants I490T [12] and S978P [12]) and also in patients diagnosed with inflammatory liver diseases, such as SC (and LPAC; R545C [13, 14]) and Biliary Cirrhosis (BC; D243A [14], K435T [14] and G535D [15]), both of which are considered to predispose to the development of liver cancer [16–22]. The animal studies and these case reports suggest that ABCB4 deficiency can predispose to (at least a subset of) severe inflammatory liver diseases, but direct evidence of a deleterious effect of the non-synonymous changes at the protein level is lacking. Here, we address for the first time whether any of these six variants affect the stability, sub-cellular localisation or function of ABCB4, and thus are likely to cause cholestasis that predisposes to secondary chronic inflammation that would be required to drive the development of SC, BC, and liver cancer. Characterisation of these clinically relevant variants also provides novel insights into the molecular mechanism of action of ABC transporters. These are discussed in relation to the available molecular models and current theories of the transport cycle.

## Materials and methods

### Plasmids

Vector: pcDNA3-ABCB4, pcDNA3-ABCB4^E558Q^, pCINeo-ATP8B1, and pCINeo-CDC50A were described previously [23]. pcDNA3-ABCB4^D243A^, pcDNA3-ABCB4^K435T^, pcDNA3-ABCB4^I490T^, pcDNA3-ABCB4^G535D^, pcDNA3-ABCB4^R545C^, and pcDNA3-ABCB4^S978P^ were generated by site-directed mutagenesis from pcDNA3-ABCB4.

### Mutagenesis

Mutagenesis was performed using QuickChange-II XL Site-Directed Mutagenesis (Stratagene, San Diego, CA, USA). In each case, introduction of the clinically relevant SNP and the absence of additional mutations were verified by sequencing of the entire cDNA and promoter.

### Mutagenic oligonucleotides

D243A, 5′-CTCTCGGCATTTAGTGCCAAAGAACTAGCTGCTTATGC-3′.

K435T, 5′-GGAAGTAGTGGCTGTGGG ACGAGCACAACGGTCCAGCTG-3′.

I490T, 5′-GTTTTCCACCACAATTGCTGAAAATACTTGTTATGGCCGTG-3′.

G535D, 5′- GCCCAGCTGAGTGATGGGCAGAAGCAG-3′.

R545C, 5′-GGATCGCCATTGCATGTGCCCTGGTTCGC-3′.

S978P, 5′-CAGAGATGTTATTCTGGTGTTTCCTGCAATTGTATTTGGTGCA-3′.

### Culture conditions

HEK293T cells were grown as monolayers in Dulbecco’s modified eagle medium (DMEM) without phenol red but supplemented with 10% (v/v) heat-inactivated foetal calf serum, 100 U ml^−1^ penicillin, 0.1 mg ml^−1^ streptomycin, 0.584 g/L L-glutamine, and 1 mM sodium pyruvate, under 5% CO_2_ at 37 °C with a water vapour saturated atmosphere (Galaxy 170 S; New Brunswick Scientific, Edison, NJ, USA).

### Preparation of crude cell lysates and Western analysis

HEK293T cells (4.5 × 10^5^) were seeded as a monolayer on 6-well plates (ThermoFisher Scientific, Waltham, MA, USA) in 2 ml medium 24 h prior to transfection. The transfection mixture (7.5 μg total plasmid DNA in 0.5% (w/v) glucose mixed with 1.5 μl polyethyleneimine (PEI) and adjusted to a final volume of 10 μl with sterile water) was prepared in individual 1.5 ml eppendorf test tubes (Eppendorf, Hamburg, Germany) and incubated for 10 min at room temperature (RT). PEI was prepared by dissolving 45 mg PEI in 8 ml sterile water, corrected to pH 7.2 with dilute HCl, passed through a sterile filter (0.2 μm) and kept at RT. For transfections with plasmid DNA encoding only wild-type (WT) or variant ABCB4, the transfection mixture contained 2.5 μg of ABCB4-encoding plasmid and 5 μg of empty pCINeo vector. For transfections with three different plasmids encoding ATP8B1 and CDC50A plus either WT or variant ABCB4, the transfection mixture contained 2.5 μg of each plasmid. After 10 min incubation, 2 ml fresh medium, pre-warmed to 37 °C, was added to each tube and the mixture applied directly to the cells. Under these conditions, we have shown previously that 60% of the cells in the population are transfected and that 96% of these cells that take up one plasmid, take up all three [24]. Transfected cells were grown for 48 h before monolayers were washed twice with phosphate buffered saline (PBS) and harvested in lysis buffer (150 mM NaCl, 20 mM HEPES pH 7.4, 1% SDS), 1 × EDTA-free complete protease inhibitor cocktail (Roche, Basel, Switzerland), 1 mM PMSF (Sigma, St Luis, MO, USA). Following denaturation in Laemmli sample buffer (5 min, 70 °C), 2 μg of each crude lysate was separated by SDS–PAGE and transferred to polyvinylidene difluoride membrane (PVDF, Millipore, Billerica, MA, USA) overnight at a constant voltage (20 V). Blots were probed with either mouse anti-ABCB4 diluted 1 in 5,000 (monoclonal P_3_II-26, Sigma, St Louis, MO, USA) or mouse anti-β-tubulin diluted 1 in 6,000 (SourceBioscience, Nottingham, UK). Binding of P_3_II-26 and anti-β-tubulin was then detected using goat anti-mouse secondary, conjugated to horseradish peroxidase diluted 1 in 2,000 (Santa Cruz, Dallas, TX, USA), for visualisation by chemiluminescence (ECL; GE Healthcare, Little Chalfont, UK). Exposed X-ray film (GE Healthcare, Little Chalfont, UK) was then scanned to allow quantitative analysis of protein abundance by densitometry using the Fiji software suite [25].

### Protein deglycosylation

For removal of N-linked glycans, crude lysate from triple-transfected cells (5 μg total protein for WT samples and 20 μg for ABCB4^R545C^ samples) was incubated for 10 min at 100 °C in the presence of denaturant (0.5% SDS, 40 mM DTT) before buffering with NaH_2_PO_4_ [to a final concentration of 50 mM, pH 7.5 (at 25 °C)], and addition of NP-40 (to 1% final concentration). Deglycosylation was carried out with 500U of PNGase F (New England Biolabs) for 2 h at 37 °C, before separation of protein by SDS–PAGE and Western analysis, probed with the C219 antibody (Dako Ltd UK).

### PC-efflux assay

Six-well plates were treated with 1 ml Poly-L-lysine for 1 h at RT and washed three times in 1 ml Dulbecco’s phosphate buffered saline (DPBS). HEK293T cells (4.5 × 10^5^) were seeded on the pre-treated plates 24 h prior to transfection. The cells were always triple transfected to co-express ATP8B1, CDC50, and ABCB4 wild-type or variant (as described above). Twenty-four hours post-transfection, the cells were fed 2 μCi [*methyl-* ^*3*^ *H*]choline (PerkinElmer, Waltham, MA, USA) and cultured for 24 h. The medium was removed and cells were washed three times in 1 ml fresh medium pre-warmed to 37 °C, and then incubated in 2 ml medium supplemented with 2 mM sodium taurocholate hydrate (TC) (Sigma, St Luis, MO, USA). After 24 h incubation, 50 μl of culture media from each well were analysed for radioactivity content in a Beckman LS 6000SC scintillation counter (Beckman Coulter, Fullerton, CA, USA). The cells attached to the dish were washed three times in 1 ml DPBS and lysed in 2 ml 0.5% (v/v) Triton-X 100. An aliquot (50 μl) from each lysate was analysed for radioactivity to determine the cellular radioactive content. PC efflux was calculated as PC detected in the media as a percentage of the total (media plus lysate), then normalised against an internal non-functional control; the Walker B mutant, ABCB4^E558Q^.

### Sub-cellular localisation of ABCB4

Sterile coverslips were placed into each well of a 12-well plate (ThermoFisher Scientific, Waltham, MA) and treated with 100 μl Poly-L-Lysine for 1 h at RT followed by three washes in 1 ml DPBS. The wells were then seeded with HEK293T cells (1.5 × 10^5^) in 1 ml medium 24 h prior to transfection. On the day of transfection, the cells were triple-transfected as described above but scaled down to the 12-well format. Forty-eight hour post-transfection, the medium was aspirated from the wells, the cells were washed three times in 1 ml PBS and then fixed with ice-cold 10% (v/v) acetone in ethanol, for 20 min at RT. Following three washes in PBS, the cells were blocked for 1 h at RT in 1 ml of 5% (w/v) BSA (bovine serum albumin; Sigma, St Louis, MO, USA) in PBS. The cells were washed three times in 1 ml PBS before incubating the coverslips with 50 μl of the anti-ABCB4 antibody, P_3_II-26 [50 μg ml^−1^ in 1% (w/v) BSA in PBS (Sigma, St Louis, MO, USA)] and/or anti-Na^+^/K^+^-ATPase antibody H-300 [50 μg ml^−1^ in 1% (w/v) BSA in PBS (Santa Cruz, Dallas, TX, USA)] for 1 h at RT. Following three washes in 1 ml PBS, the coverslips were incubated with 50 μl of Alexa Fluor® 568 Dye-conjugated goat anti-mouse IgG and/or Alexa Fluor® 488 donkey anti-rabbit IgG [2 μg ml^−1^ (Invitrogen, Carlsbad, CA, USA) in 1% (w/v) BSA in PBS] for 1 h at RT. Nuclei were stained in the same incubation period with 1.5 ng μl^−1^ DAPI (4′,6-diamidino-2-phenylindole; Invitrogen, Carlsbad, CA, USA). The coverslips were then washed three times in 1 ml PBS and mounted onto microscope slides using FluorSave^™^ Reagent (Millipore, Billerica, MA, USA). Cells were viewed using a Zeiss LSM710 confocal laser scanning microscope based on an Axiovert inverted microscope (Carl Zeiss, Oberkochen, Germany) with a 63 × oil immersion objective and a numerical aperture of 1.4. Laser lines 488, 568, and 405 nm were used to excite Alexa Fluor® 488/568 and DAPI, respectively. The pinhole was set to 1 Airy unit at 2.0% laser power for all experiments. Images were acquired with sequential scanning to allow cross-talk free Alexa Fluor® 488/568 and DAPI images to be collected. Images were depixelated and background was subtracted before co-localisation analyses (coloc 2) in the Fiji software suite.

## Results

### Expression levels of the ABCB4 variants in HEK293T cells and a first indication of functionality

**Fig. 1:**
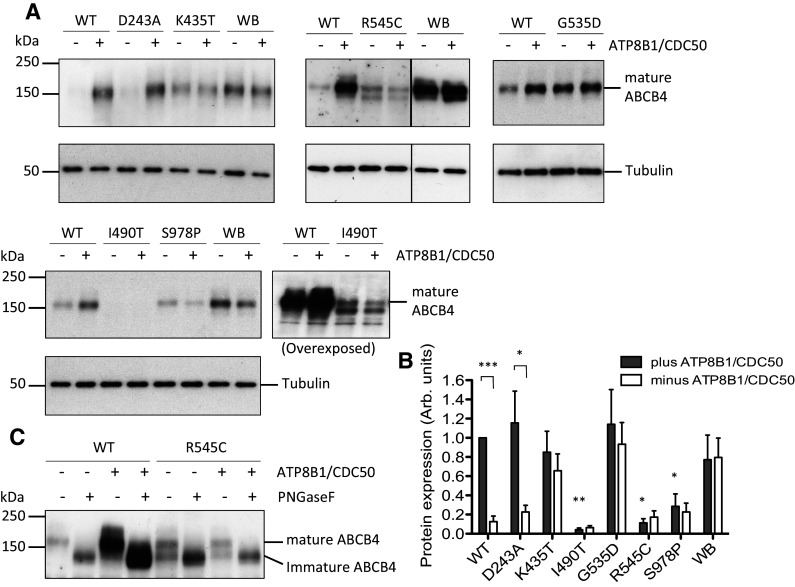
Expression of ABCB4 variants in the presence and absence of ATP8B1/CDC50 and glycosylation status of the R545C variant. Abundance of ABCB4 variants D243A, K435T, G535D, R545C, I490T, and S978P was examined by Western analysis of whole-cell lysates from transiently transfected HEK293T cells, in the presence and absence of ATP8B1/CDC50. **a** Representative Western blots showing the expression levels of the variants with respect to the wild-type (WT) transporter and the catalytically inactive Walker B mutant (WB). ABCB4 was detected by monoclonal antibody P_3_II-26. Tubulin was used as a loading control. **b** Biological replicates (*n* ≥ 3) were digitised and quantified by densitometry. The plot shows mean arbitrary units of expression level + s.e.m. normalised to the internal wild-type control (WT in the presence of ATP8B1/CDC50). Statistical analysis was by unpaired Student’s *t* test and compares expression level in the presence and absence of ATP8B1/CDC50, showing significant difference for WT and D243A samples. The expression levels of I490T, R545C, and S978P are also statistically lower than the level of wild-type ABCB4 in cells expressing ATP8B1/CDC50 (*n* ≥ 3; ***p < 0.001, **p < 0.01, *p < 0.05). **c** Aliquots (5 and 20 μg total protein for the WT and R545C samples, respectively) were subjected to PNGase F digestion or left untreated, as indicated. Western was probed with monoclonal anti-ABCB4 antibody C219

Wild-type ABCB4 is cytotoxic to HEK293T cells and expresses very poorly by itself. Expression can be rescued and the cytotoxicity reduced to background levels by co-expression of the phosphatidylserine flippase ATP8B1/CDC50 [23]. We have shown previously that the deleterious effect on the host cell is a direct consequence of ABCB4 PC-floppase activity [23, 24], which is why the inactive Walker B mutant ABCB4^E558Q^ that lacks the catalytic base required for ATP hydrolysis, expresses to a high-level irrespective of the ATP8B1/CDC50 status (Fig. 1a, b). Western analysis can, therefore, be a useful first indication of the effect of non-synonymous mutations, providing information on protein abundance (a combination of translation, and protein folding and stability) and of function (because high-level expression of ABCB4 variants in the absence of ATP8B1/CDC50 suggests low PC-efflux activity). The D243A, K435T, G535D, R545C, I490T, and S978P variants were engineered into pcDNA3-ABCB4 to mimic the SNPs reported in patients. HEK293T cells were transfected transiently, with either three plasmids encoding ABCB4 (the wild-type (WT) transporter or a variant or the catalytically inactive ABCB4^E558Q^ mutant) plus ATP8B1 and CDC50, or encoding an ABCB4 plus two aliquots of empty vector.

The expression levels of the variants were assessed by Western analysis in the presence and absence of ATP8B1/CDC50 and the abundance of each was quantified and compared to that of the wild-type and ABCB4^E558Q^ proteins (representative blots are shown in Fig. 1a and replicate data sets are quantified in Fig. 1b). The novel variants linked to inflammatory liver diseases displayed a range of phenotypes. The two variants directly linked to liver cancer, ABCB4^I490T^ and ABCB4^S978P^, expressed at reduced levels, 6 and 22%, respectively, relative to the expression of the WT transporter in the presence of ATP8B1/CDC50. The ABCB4^I490T^ signal needed longer exposure time to visualise and a more complex pattern was evident with two prominent small molecular weight species in addition to the full-length 160 kDa species (for the purpose of quantification, only the level of the mature 160 kDa species was measured, see below for explanation). Co-expression of ATP8B1/CDC50 had no statistically significant effect on the expression level of these variants. Of the variants linked to the development of biliary cirrhosis, ABCB4^D243A^ has an expression profile that is indistinguishable statistically from that of the wild-type transporter, while ABCB4^K435T^ and ABCB4^G535D^ expressed to equally high levels in the presence and absence of ATP8B1/CDC50 and could not be distinguished statistically from the catalytically inactive ABCB4^E558Q^ mutant. The variant linked to the development of sclerosing cholangitis, ABCB4^R545C^, expressed at a lower level and migrated primarily as two protein species on the gel (the abundance of the larger, 160 kDa, species measured 17% of the wild-type transporter). Enzymatic deglycosylation of the sample resolves the two species into a single species that migrates with the same mobility as the deglycosylated wild-type transporter (Fig. 1c). The two species of ABCB4^R545C^ therefore differ in glycosylation status suggesting that the faster migrating species is immature protein (probably in the endoplasmic reticulum) and the slower migrating 160 kDa species is the fully glycosylated mature protein that could be present anywhere from the terminal cisternae of the Golgi apparatus where the glycans are matured, to the plasma membrane or recycling vesicles.

### ABCB4^D243A^, ABCB4^G535D^, ABCB4^K435T^, and ABCB4^S978P^ localise primarily to the plasma membrane, but significant fractions of ABCB4^I490T^ and ABCB4^R545C^ are retained intracellularly

**Fig. 2:**
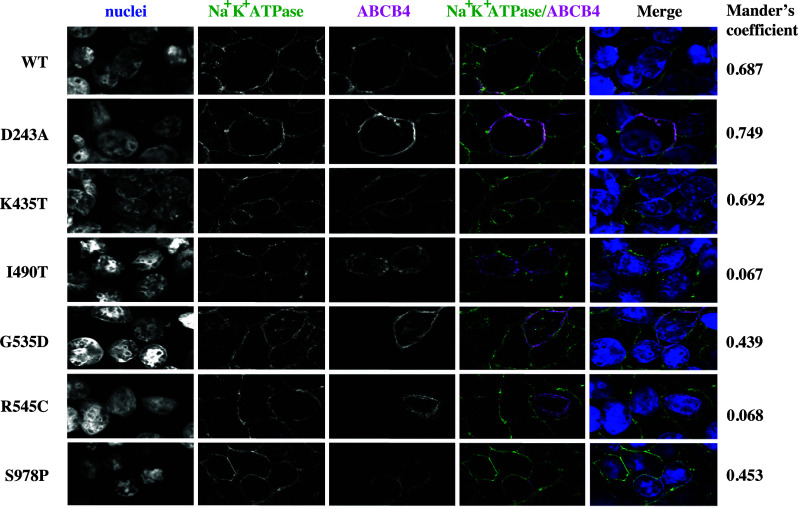
Confocal analysis of ABCB4 variant sub-cellular localisation. Trafficking of ABCB4 mutants D243A, K435T, G535D, R545C, I490T, and S978P was examined by confocal microscopy (shown in magenta in the merged images) in cells co-expressing ATP8B1/CDC50. The Na^+^/K^+^ ATPase, shown in green in the merged images, marks the plasma membrane. Co-localisation in the merged image is white when the pixels for the Na^+^/K^+^ ATPase and ABCB4 overlap and have the same intensity. The Mander’s coefficient for the given field of view provides a measure of this co-localisation above a calculated threshold, irrespective of pixel intensity

Cells were transiently transfected to express ABCB4 and ATP8B1/CDC50 then fixed, permeabilised and stained for ABCB4, and also the Na^+^/K^+^ ATPase as a plasma membrane marker. Confocal microscopy showed that the WT protein traffics efficiently to the plasma membrane where it co-localises with Na^+^/K^+^ ATPase giving a high Mander’s coefficient (0.687), with little signal detected intracellularly (Fig. 2). ABCB4^D243A^ and ABCB4^K435T^ also show evidence of co-localisation with Na^+^/K^+^ ATPase with similar Mander’s coefficients to the wild-type transporter. Together with the Western blot data which show similar expression levels for the 160 kDa mature species of the wild-type and these variants, it seems reasonable to conclude that ABCB4^D243A^ and ABCB4^K435T^ are present in the plasma membrane at similar levels to the wild-type transporter. ABCB4^G535D^ and ABCB4^S978P^ also have high Mander’s coefficients of 0.439 and 0.453, respectively for overlap with the Na^+^/K^+^ ATPase, suggesting that almost half of the mature protein observed on their respective Western blots is localised to the plasma membrane. In contrast, ABCB4^I490T^ and ABCB4^R545C^ were observed largely in an intracellular compartment, with a little overlap with the plasma membrane marker (Mander’s coefficients of 0.067 and 0.068, respectively). This is consistent with the presence of lower molecular weight species of both variants in Western blot. The lack of mature glycans on the lower molecular weight species of ABCB4^R545C^ (Fig. 1c) is consistent with a staining pattern localised primarily to the nuclear periphery suggesting that this variant folds inefficiently and is largely retained within the ER. Although we have not shown that ABCB4^I409T^ is differentially glycosylated, we tentatively suggest from the staining pattern that this variant is also likely to fold inefficiently and may be targeted for degradation via the ER-associated degradation pathway resulting in the low levels detected on Western blot (Fig. 1a).

### ABCB4^D243A^ is partially active, ABCB4^K435T^ and ABCB4^G535D^ are inactive, and efflux of PC from cells expressing ABCB4^I490T^, ABCB4^R545C^ and ABCB4^S978P^ was not detected

**Fig. 3:**
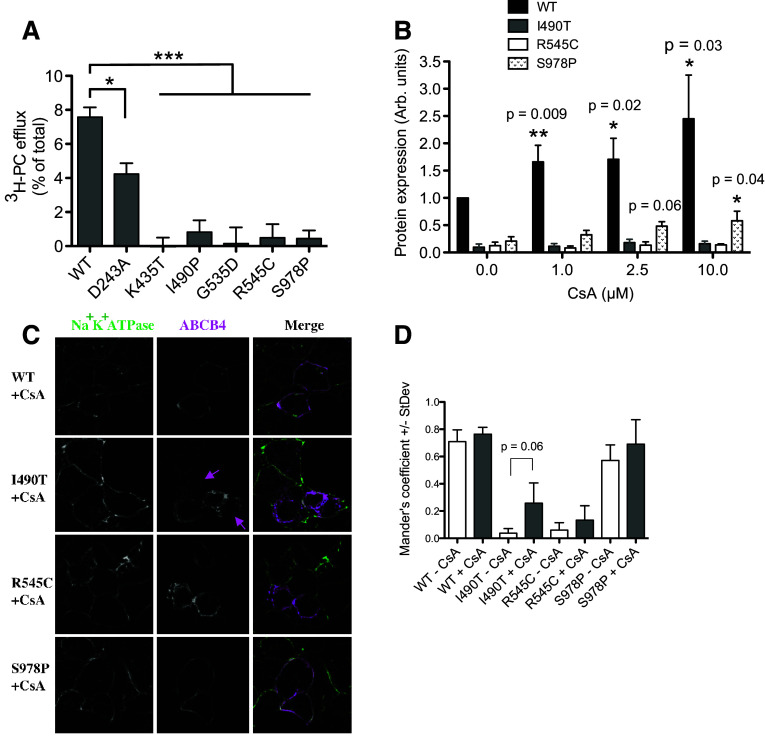
PC-efflux activity of ABCB4 variants and response of variants with poor proteostasis characteristics to a molecular chaperone. **a** Cells expressing ATP8B1/CDC50 and ABCB4 (wild-type or variant) were fed tritiated choline to measure PC efflux. Background PC flux has been accounted for by subtraction of the level extracted from cells expressing the catalytically inactive Walker B mutant E558Q. Mean efflux activity + s.e.m. are plotted. Statistical analysis was by the one-way analysis of variance with Tukey’s multiple comparison test to compare the efflux activity to the wild-type ABCB4 (*n* ≥ 4; ***p < 0.001, **p < 0.01, *p < 0.05). **b** Cells expressing ATP8B1/CDC50 and ABCB4 (wild-type or variants ABCB4^I490T^, ABCB4^R545C^ and ABCB4^S978P^) were treated with CsA, as indicated. Western analysis was used to assess the ability of CsA to rescue the variant phenotypes. Mean expression level + s.e.m. are plotted. Statistical analysis was by unpaired Student’s *t* test and compares the expression level to the equivalent ABCB4 in the presence of vehicle (DMSO) but absence of CsA (*n* = 4; *p < 0.05, **p < 0.01). **c** Sub-cellular localisation of I490T, R545C, and S978P in cells treated with 10 μM CsA from 24 h post-transfection was analysed by confocal microscopy. ABCB4 is shown in *magenta* and the Na^+^/K^+^ ATPase is shown in *green* as a marker of the plasma membrane. Faint staining of ABCB4^I490T^ at the periphery of cells (not seen in the absence of CsA) is indicated by the magenta arrows. **d** Mander’s coefficients for overlap of ABCB4 (*magenta* pixels) with the Na^+^/K^+^ ATPase (*green* pixels) independent of pixel intensity were calculated for three fields-of-view

HEK293T cells were transfected to express ATP8B1/CDC50 plus wild-type or variant ABCB4. Twenty-four hour post-transfection, the cells were fed ^3^H-choline to convert into ^3^H-PC. Previously, we have shown that cellular efflux of PC is dependent on bile salts, taurocholate was therefore added 48 h post-transfection to extract the flopped radiolabelled PC from the outer leaflet of the plasma membrane. PC is a normal component of plasma membranes and is enriched in the outer leaflet. ^3^H-PC can therefore be extracted from naïve cells which do not express ABCB4, or from cells expressing the catalytically inactive Walker B mutant ABCB4^E558Q^. However, significantly more ^3^H-PC is extracted from cells expressing wild-type ABCB4 due to the activity of the transporter (Fig. 3a). ^3^H-PC efflux via ABCB4^D243A^ was also significantly above background but consistently less than from the wild-type transporter. In paired experiments, ^3^H-PC efflux from cells expressing ABCB4^D243A^ was on average 56%+/−0.16% of the wild-type level. For ABCB4^K435T^ and ABCB4^G535D^, there was no evidence of any ^3^H-PC efflux, as expected from the Western data, because the high expression levels of these variants in the absence of ATP8B1 and CDC50 implied a lack of cytotoxicity and, therefore, a lack of transport activity. With ABCB4^K435T^ and ABCB4^G535D^ trafficking to the plasma membrane relatively efficiently, it is evident that these variants are mechanistically impaired. No ^3^H-PC-efflux activity was observed for ABCB4^I490T^, ABCB4^R545C^, and ABCB4^S978P^. The levels of ^3^H-PC extracted by the added taurocholate were significantly reduced compared to that extracted from cells expressing the WT protein (Fig. 3a), but not significantly different from the background level from the E558Q Walker B mutant cells. It is possible that the low levels of expression of these variants, in particular for ABCB4^I490T^ and ABCB4^R545C^ for which significant fractions appear immature and/or retained intracellularly, fall outside the sensitivity of the functional assay and that function could be rescued by improving their proteostasis properties.

### The molecular chaperone cyclosporin A increases the stability of ABCB4^S978P^ and wild-type ABCB4, but has no effect on the abundance of ABCB4^I490T^ or ABCB4^R545C^

Previously, cyclosporin A (CsA) has been shown to improve the folding kinetics of mutants of the multidrug efflux pump ABCB1, leading to improved stability, increased trafficking to the plasma membrane, and increased function [26]. We have shown that CsA can also stabilise the expression of specific ABCB4 mutants, albeit at the cost of irreversible PC-efflux inhibition [24]. The primary phenotype of ABCB4^I490T^, ABCB4^R545C^, and ABCB4^S978P^ is low abundance. To test if their expression levels could also be rescued by CsA, ATP8B1, CDC50, and ABCB4 encoding the variant or wild-type transporters were transiently expressed in HEK293T cells. Twenty-four hours post-transfection, the cells were incubated with increasing concentrations of CsA. After a further 24 h, the cells were harvested for Western analysis. None of the ABCB4 mutants were fully rescued to the wild-type level of expression, but ABCB4^S978P^ expression was improved, trending upward in a dose-dependent manner to achieve a statistically significant three-fold increase with 10 μM CsA (Fig. 3b). This provides proof of principle that intervention to improve the abundance of ABCB4^S978P^ in patient livers might alleviate cholestatic symptoms. The abundance of ABCB4^I490T^ and ABCB4^R545C^ was insensitive to CsA. However, the increase in stability of the wild-type protein in response to the drug shows that molecular chaperones could also be efficacious in patients heterozygous at the *ABCB4* locus with the wild-type allele.

### Cyclosporin A has a subtle effect on the localisation of ABCB4^I490T^ at the plasma membrane

CsA has previously been shown to increase the localisation of the ABCB4^A935D^ mutant (a cause of PFIC3) to the plasma membrane without increasing its abundance in the cell [24]. We therefore tested whether CsA would affect the sub-cellular localisation of ABCB4^I490T^, ABCB4^R545C^ or ABCB4^S978P^. A subtle increase in the Mander’s coefficient for overlap with the Na^+^K^+^ATPase was observed, in particular, for ABCB4^I490T^ in the presence of 10 μM CsA (Fig. 3c); however, this did not reach statistical significance when multiple fields were analysed (Fig. 3d). Wild-type ABCB4 in the presence and absence of CsA is shown for comparison. Importantly, the >twofold increase in expression of ABCB4^WT^ following treatment with CsA (Fig. 3b) has not negatively affected the Mander’s coefficient (Fig. 3d), indicating that the additional protein is located at the plasma membrane.

## Discussion

Mice deficient in *Abcb4* develop liver cancer as a result of chronic inflammation. In humans, non-synonymous SNPs in *ABCB4* have been identified in patients with liver cancer (cholangiocarcinoma; Table 1) [12]. In both of the patient case studies, relatives heterozygous for the same I490T and S978P variants presented with cholestatic diseases that are more commonly associated with ABCB4 dysfunction, suggesting that pre-existing cholestasis may also predispose to cancerogenesis in humans. Population genetic studies have also linked ABCB4 variants to the development of inflammatory cholangiopathies, sclerosing cholangitis, and cirrhosis [14] that are already considered to predispose to cholangiocarcinoma [16–18, 21, 22]. (It is important to stress here that the population genetics have shown that variation at the *ABCB4* locus is not a strong determinant of primary sclerosis cholangitis or primary biliary cirrhosis, but the SNPs encoding D243A, K435T, and R545C were observed only in the patient cohort and not in the healthy controls. Others have already speculated that this may reflect a heterogeneous etiology of these conditions in which a subset is linked to ABCB4 deficiency [27]). A further case study also linked the G535D variant to development of cirrhosis in a patient who also has a family history of cholestatic disease (Table 1) [15]. Combining what is already known about the role of ABCB4 in bile formation and flow, with the animal data and human genetic data, it seems plausible that *ABCB4* SNPs underlie the inflammatory liver disease in these patients. What is missing is evidence of cause and effect at the level of the transporter. We, therefore, engineered the SNPs into the *ABCB4* cDNA to test whether the non-synonymous amino-acid changes negatively affect transporter expression and/or function and expressed these transiently in a human cell line. A hepatocyte cell line would not have been appropriate for this purpose because of the co-ordinate regulation of the bile flow transporters and their potential induction by the need to add bile salts to solubilise the flopped PC. HEK293T cells were selected as the host cell because they lack endogenous ABCB4 expression and are readily transfected (very high levels of co-transfection were necessary to express ATP8B1 and CDC50 with ABCB4 in the same cells). While the HEK293T cells express wild-type ABCB4 efficiently at the plasma membrane, they have limitations as a host cell, because they not polarised and may lack hepatocyte-specific factors that could affect the protein folding and trafficking landscape.

**Table 1:** Patient histories and protein phenotypes of the *ABCB4* SNPs linked to inflammatory liver disease

SNP	Protein	References	Sex	Zygosity	History	Amino-acid position	Abundance	Localisation	PC-efflux activity
A728C	D243A	[14]	Unknown	Unknown	BC	TMD1 TMH4	Normal	pm	Half
A1304C	K435T	[14]	Unknown	Unknown	BC	NBD1 Walker A motif	Normal	pm	Inactive
T1469C	I490T	[12]	Male	Heterozygous	Carcinoma	NBD1 α-helical subdomain	Low (6%)	Intracellular	Unknown^c^
T1469C	I490T	[12]	Female^a^	Heterozygous	CG, Jaundice, Pruritis				
G1606A	G535D	[15]	Female	Heterozygous	CG, ICP, BC	NBD1 ABC signature motif	Normal	pm	Inactive
G1606A	G535D	[15]	Female^a^	Heterozygous	ICP				
C1633T	R545C	[14]	Unknown	Unknown	SC	NBD1 α-helical subdomain	Low (17%)	Intracellular	Unknown^c^
T2932C	S978P	[12]	Female	Heterozygous	Carcinoma	TMD2 TMH12	Low (22%)	pm	Unknown^c^
T2932C	S978P	[12]	Female^b^	Heterozygous	LPAC, ICP				

The population genetic study of Pauli-Magnus et al. [14] describes the diagnoses as Primary Sclerosing Cholangitis and Primary Biliary Cirrhosis, although we would argue that the conditions are likely secondary to underlying inflammatory cholestasis in these patients

*CG* cholesterol gallstones (cholelithiasis), *BC* biliary cirrhosis, *SC* sclerosing cholangitis, *ICP* intrahepatic cholestasis of pregnancy, *LPAC* low phospholipid-associated cholelithiasis

^a^Daughter of the patient above

^b^Sister of patient above

^c^Cells expressing these mutants do not efflux significant levels of PC, but this could due to a lack of the transporter at the plasma membrane (pm), rather than the inactivity of the protein

All of the variants significantly impaired the ability of ABCB4 to secrete PC from cells but their phenotypes at the protein level were distinct (Table 1). Prior work on different SNPs indicated that *ABCB4*-haploinsufficiency can result in clinical symptoms, and the *in vitro* activity of the mutant floppases correlated well with disease severity after allelic heterogeneity was accounted for [24]. Using these data as a prognostic barometer, individuals with half the PC-floppase activity (the expected level of activity in patients with ABCB4^K435T^, ABCB4^I490T^, ABCB4^G353D^, ABCB4^R545C^, or ABCB4^S978P^ in heterozygosity with the wild-type allele) would be expected to present with clinical cholestasis. Clinical symptoms are also evident in patients with a genotype that equates to 75% of the normal PC-floppase activity (ABCB4^wild−type^/ABCB4^S320F^). The previously characterised ABCB4^S320F^ was found to be fully active, but expression was reduced by half, compared to the WT. ABCB4^D243A^ is similar, because, although its abundance is normal, it only flops half the PC of the WT transporter. It is reasonable to expect, therefore, that the *ABCB4* genotype of the patients in the current study causes a chronic cholestatic phenotype due to canalicular bile salt micelles that are low in PC. The ensuing damage to the biliary tree would likely produce an inflammation response that drives secondary sclerosis or cirrhosis, and predisposes to liver cancer [28, 29], as observed in the animal models [30–32].

### Mechanistic implications

**Fig. 4:**
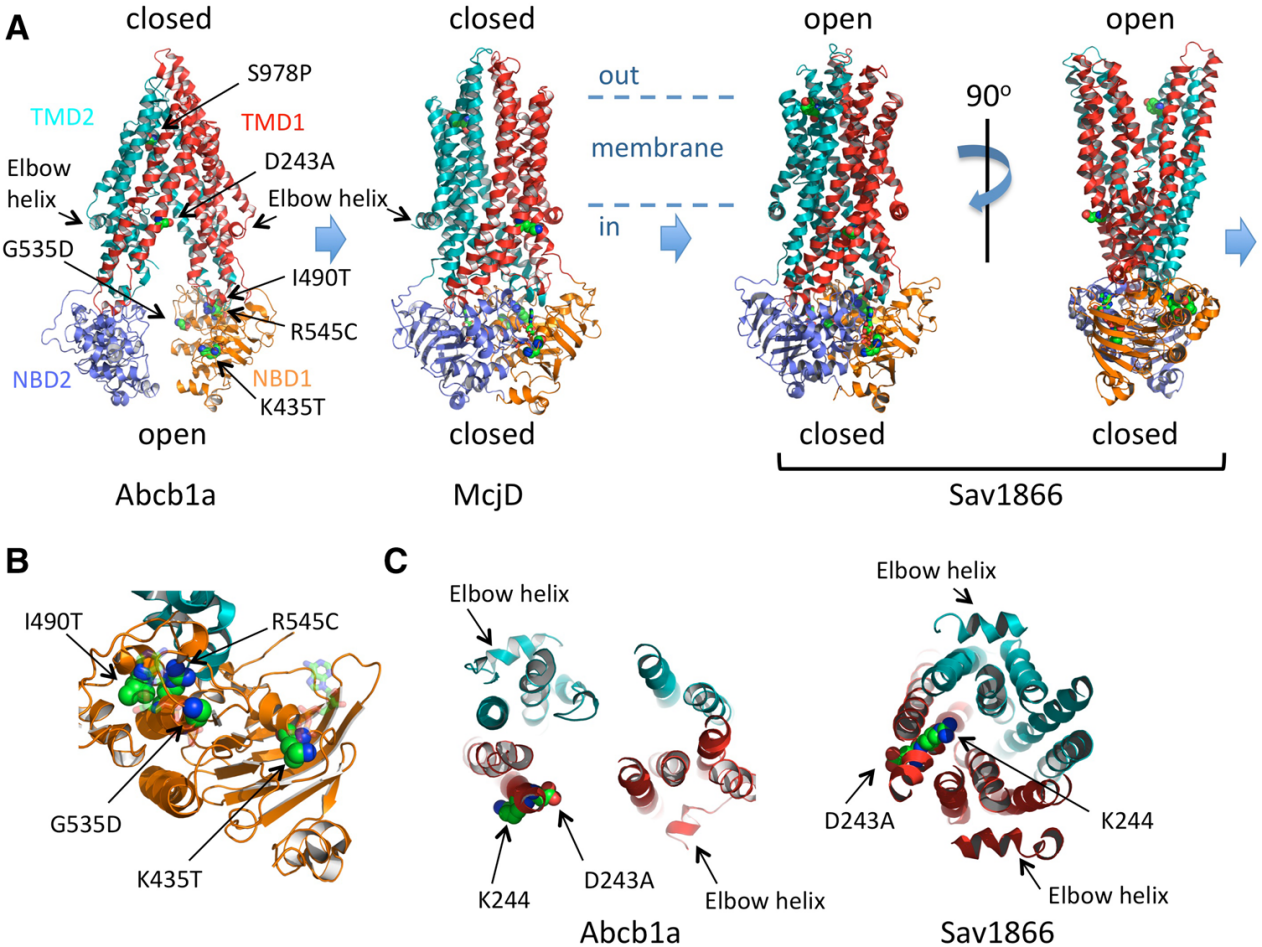
Molecular modelling of the impact of the non-synonymous amino-acid changes on ABC transporter fold and function. Cartoon representation of three conformations of ABC exporters observed in X-ray crystallographic studies. The inward-open state of Abcb1a (pdb 3G5U [33]) is nucleotide-free and considered to be the high affinity state for binding transport substrate. The occluded, closed state of McjD (pdb 4p10 [34]), crystallised with two molecules of non-hydrolysable ATP analogue shown in ball and stick form, is considered to represent an intermediate state of the transport cycle with transport substrate and ATP bound. The outward-open state of Sav1866 (pdb 2HYD [35]), crystallised with two molecules of ADP shown in *ball* and *stick* format (although the NBD:NBD interface is considered to represent the ATP-bound state), is thought to depict the conformation after release of the transport substrate but prior to ATP hydrolysis. ATP hydrolysis and ADP and phosphate release should restore the transporter to the Abcb1a conformation. In all models, the equivalent residues (identified by alignment of the primary sequences by Clustal W) to ABCB4 D243, K435, G535, I490, R545, and S978 are shown as spheres and coloured elementally. The likely position of the membrane is indicated, with the head groups of the inner leaflet membrane lipids just above the elbow helices. **b** Close-up view of NBD1 (based on McjD) from the perspective of NBD2 showing the position of K435 in the Walker A motif, I490 and R545 in the α-helical subdomain and G535 in the ABC signature motif of the α-helical subdomain. The ATPs are shown in *transparent ball* and *stick* format. NBD2 and TMD2 are hidden for clarity. **c** A 30 Å slab viewed from above the plane of the membrane, showing the TM helices at the intracellular face of the membrane and the changing position of D243 and K244 as the transporter moves from the *inward-open* (*Abcb1a*) to the *outward-open* (*Sav1866*) conformation

Both ABCB4^K435T^ and ABCB4^G535D^ express well irrespective of ATP8B1/CDC50 status and localise to the plasma membrane but lack PC-floppase activity. Both of these residues are in highly conserved motifs within the first nucleotide-binding domain (NBD1) of ABCB4 (Fig. 4a, b). K435 is part of the Walker A motif (GxxGxGK^435^ST) and G535 is part of the ABC signature motif (LSG^535^GQ) that are critical for the binding of ATP at the interface between the two NBDs. The two motifs affect different ATP-binding pockets (Fig. 4b), because two molecules of ATP are co-ordinated at the interface between the Walker A motif of one NBD and the ABC signature of the other NBD. Current thinking on the transport cycle of an efflux ABC transporter is shown in Fig. 4a as the transition between three conformational states (derived from the models of three different ABC transporters, each crystallised in a different conformation). Interpreting these models with respect to ABCB4, the inward-open state depicted by model of Abcb1a on the left should bind PC from the inner leaflet of the membrane (the so-called elbow helix is considered to be cytosolic but juxtaposed to the head groups of the membrane lipids of the inner leaflet). The binding of PC would be hypothesised to induce the conformational change to the closed state (the model of McjD) which requires two ATPs to be bound at the interface between the NBDs. This fully closed state is likely to be transient, progressing to the outward-open conformation (Sav1866 model; although crystallised with two ADPs at the NBD:NBD interface, it is generally considered to reflect the ATP-bound conformation). With respect to ABCB4, we speculate that the outward-open conformation releases PC (into the outer leaflet, or directly into a bile salt micelle) and ATP hydrolysis would drive the transporter back into the inward-open conformation. Phenotypically, the K435T and G535D variants appear similar to the catalytically inactive Walker B mutant E558Q, but, mechanistically, they are likely to interrupt the transport cycle at an earlier step, because they would be expected block ATP binding to one or the other binding pocket and so prevent the transition between the inward-open and inward-closed states, whereas the Walker B mutant would block the return of the outward-open state to the inward-open state by preventing ATP hydrolysis.

The I490T and R545C variants of ABCB4 express only to low levels compared to the wild-type transporter and are largely retained intracellularly. Extrapolating from the available structural data, I490 and R545 are located within 5 Å of each other in the tightly folded alpha-helical subdomain that forms the ABC signature motif (Fig. 4b). Both variants change the size and polar nature of the side chain and are therefore likely to influence the folding kinetics of the subdomain. In particular, the side chain of R545, which is in the same helix as the ABC signature motif, appears to form several hydrogen bonds that are likely important for the integrity of the motif’s structure; these would be lost in the R545C variant. Poor folding kinetics in this subdomain most likely results in the nascent protein being held in the endoplasmic reticulum before degradation by the quality control process, which would fit with the observed glycosylation differences and the low abundance of these variants at the plasma membrane.

ABCB4^D243A^ is the least debilitated of the variants. It expresses to the level of the wild-type protein, and localises to the plasma membrane. It also remains cytotoxic to the HEK293T cells in the absence of ATP8B1/CDC50, but it is not fully functional and only secretes 56% of the PC compared to the wild-type transporter. D243 is located towards the intracellular end of transmembrane helix (TMH) 4 of the first TMD. In Fig. 4c, a 30 Å slab showing the transmembrane helices at the intracellular face of the inward-open and inward-closed models is shown (the elbow helices which are cytosolic are also visible within the slab, and allow ease of reference to the models in Fig. 4a). In the inward-open model (Abcb1a), the side chain of D243 is orientated towards the central cavity formed by the transporter, but in the inward-closed model (Sav1866), the equivalent side chain (a glutamate) is orientated to the periphery of the protein. In ABCB4, the adjacent residue is a lysine (K244), and in the drug efflux pumps, Abcb1a and Sav1866, the adjacent residues are lysine and arginine, respectively. We speculate that as the inward-open, central cavity is closed TMH4 rotates to move the negatively charged D243 residue out of the cavity to be replaced with a positively charged K244 residue. The choline head group of PC carries a positive charge, and the drugs transported by Abcb1a and Sav1866 often contain a single cationic group, suggesting that electrostatic attraction may be important for substrate binding within the inward-open cavity (Abcb1a model) which converts to electrostatic repulsion to drive the substrate across the membrane as the transporter changes to the outward-open conformation (Sav1866 model). Replacing the negatively charged side chain of the aspartate with the smaller neutral side chain of alanine would reduce the electrostatic potential in the binding cavity of the transporter. Further investigation will be needed to confirm this hypothesis.

S978 is also located within a transmembrane domain, towards the extracellular end of TMH12. ABCB4^S978P^ expression is low, although it traffics to the plasma membrane efficiently with very little observed intracellularly. PC flopping activity is not significantly different to the catalytically inactive Walker B mutant. S978 is conserved in Abcb1a and in the inward-open configuration its side chain is orientated towards the apex of the central cavity, where it has been shown to interact with an inhibitor of the transporter [36]. The relevance of this is unclear for PC flopping by ABCB4, but the equivalent residues (tyrosines that preserve the hydroxyl moiety of the side chain) in McjD and Sav1866 (the closed and outward-open states, respectively) are re-orientated away from the central cavity. Proline residues cannot donate hydrogen bonds and are known to disrupt alpha-helices. They are rare in TMHs where they typically introduce a kink in the helix. As ABCB4^S978P^ can be observed in the plasma membrane (with little seen in the ER), it may be that this non-functional variant with a kinked TMH12 is turned over more rapidly which could contribute to the low abundance observed.

In summary, we have demonstrated that ABCB4 SNPs encoding D243A, K435T, G535D, I490T, R545C, and S978P significantly compromise the transporter. Comparison to a prognostic scale suggests that each would cause cholestasis even if the second allele is wild-type. Indeed, all of these variants would be expected to cause PFIC3 in the homozygous state (perhaps except ABCB4^D243A^, which might, nevertheless, predispose to developing late onset PFIC3 similar to individuals homozygous for ABCB4^S320F^ [24]). Together with the genetic evidence, patient histories, and animal studies, these data at the protein level provide a compelling argument that the *ABCB4* genotype can underlie development of inflammatory liver diseases that extends to cancer. Management and treatment options should therefore include addressing the underlying cholestasis, and may be helped by future development of drugs that improve the proteostasis of the wild-type allele but do not inhibit its transport activity.
